# Regional Homogeneity Abnormalities in Early-Onset and Adolescent-Onset Conduct Disorder in Boys: A Resting-State fMRI Study

**DOI:** 10.3389/fnhum.2019.00026

**Published:** 2019-02-07

**Authors:** Wanyi Cao, Chuting Li, Jing Zhang, Daifeng Dong, Xiaoqiang Sun, Shuqiao Yao, Bingsheng Huang, Jun Liu

**Affiliations:** ^1^Medical Psychological Center, Second Xiangya Hospital, Central South University, Changsha, China; ^2^Medical Psychological Institute of Central South University, Changsha, China; ^3^Health Science Center, School of Biomedical Engineering, Shenzhen University, Shenzhen, China; ^4^Department of Radiology, Second Xiangya Hospital, Central South University, Changsha, China

**Keywords:** early-onset conduct disorder, adolescent-onset conduct disorder, regional homogeneity, resting-state functional magnetic resonance imaging, developmental taxonomic theory

## Abstract

**Purpose**: Developmental taxonomic theory posits that formation of early-onset conduct disorder (EO-CD), is considered to have a neurodevelopmental etiology and have more severe psychosocial and neuropsychological dysfunction than adolescent-onset CD (AO-CD), which is thought to stem largely from social mimicry of deviant peers. The purpose of the current study was to investigate whether regional homogeneity (ReHo), denoting the spontaneous brain activity, supports developmental taxonomic theory in a resting state (rs).

**Materials and Methods**: Rs-functional magnetic resonance imaging (fMRI) examinations were administered to 36 EO-CD patients, 32 AO-CD patients, and 30 healthy controls (HCs). All participants were male adolescents, aged between 12 and 17 years old. A one-way analysis of covariance (ANCOVA), with age and IQ as covariates, was performed to identify regions with significant group differences in ReHo values, followed by a *post hoc* analyses.

**Results**: Compared with the AO-CD groups, EO-CD had higher ReHo values in the right middle/inferior frontal gyrus. Compared with the HCs, the EO-CD group exhibited lower ReHo values in the left precuneus, left middle occipital gyrus, left cerebellum posterior lobe and the right inferior parietal lobule, as well as higher ReHo values in the right middle frontal gyrus, left insula/inferior frontal gyrus, right postcentral gyrus, and the left anterior cingulate gyrus. Compared with the HCs, the AO-CD group showed lower ReHo values in the bilateral precuneus, left cerebellum posterior lobe, and the right inferior parietal lobule.

**Conclusion**: Significant differences in ReHo were observed between the EO-CD and AO-CD groups, implying distinct neuropathological mechanisms of the two CD subtypes, consistent with developmental taxonomic theory. CD-associated abnormalities in ReHo may be related to high-order cognitive and low-level perceptual system impairments in CD.

## Introduction

Conduct disorder (CD) is a common psychiatric disorder in childhood and adolescence, characterized by persistent aggressive and antisocial behaviors, such as violating the basic rights of others, lying, stealing, and truancy (Segal, 2000). The DSM-IV distinguishes early-onset CD (EO-CD), diagnosed when CD symptoms are recognized prior to 10 years of age, from adolescent-onset CD (AO-CD), diagnosed when CD symptoms become apparent after the child’s 10th birthday (Segal, 2000).

According to the developmental taxonomic theory, it has been supposed that the etiology of EO-CD is strongly related to neurodevelopmental processes, whereas AO-CD may be a consequence of the social learning of bad behaviors from a peer group and the motivation to gain more adult privileges (Moffitt, 1993). Symptom severity, risk factors, and development path characteristics differ between EO-CD and AO-CD (Dandreaux and Frick, 2009; Silberg et al., 2014). Relative to AO-CD, EO-CD is characterized by a more severe pattern of aggressive and antisocial behaviors and a greater risk of developing antisocial personality disorder in adulthood (Moffitt et al., 2002). Additionally, EO-CD is more likely than AO-CD to be associated with neurological and cognitive deficits (e.g., executive function), temperament challenges (e.g., impulsivity), and social environmental risks (e.g., poor parenting, unstable family background, and poverty), as well as genetic vulnerability. AO-CD is related to a gap between biological maturity and social maturity, influenced by deviant peer group pressures (Moffitt, 1993).

Behavioral studies suggest that the two subtypes of CD have some dissociable neurocognitive, psychological, and physiological deficits, as reflected by the results of neuropsychological tasks, designed to assess cognitive and emotional functions (Fairchild et al., 2008, 2009, 2010). These differences point to different underlying pathophysiological mechanisms of EO-CD and AO-CD. Recent neuroimaging studies have explored differences between the brains of patients with EO-CD vs. AO-CD. A functional magnetic resonance imaging (fMRI) study of facial emotion processing, the first to compare brain activations across the two CD subtypes directly, revealed that the EO-CD group had less activation in the amygdala and superior temporal gyrus than in the AO-CD group, in the sad vs. neutral face condition (Passamonti et al., 2010). Structural MRI have explored the differences between EO-CD and AO-CD, reporting gray matter volume reductions in neural circuits involved in social emotional stimuli (Fairchild et al., 2011), thinning paralimbic cortices in AO-CD (Jiang et al., 2015). All the above studies are either structural MRI or task-based fMRI. To our best knowledge, there has been no resting-state (rs) fMRI studies investigating the difference between EO-CD and AO-CD. rs-fMRI may reflect spontaneous neural activity (Biswal et al., 1995). rs-fMRI has great advantages in clinical applications because it does not require any stimulation and response, which subjects, especially patients can perform easily. There is a need to explore whether EO-CD and AO-CD present similar or distinct characteristics of spontaneous brain activity in a resting state.

In rs-fMRI, subjects keep their eyes either closed or fixated on a crosshair during scanning in a task-free paradigm. Zang et al. (2004) developed the regional homogeneity (ReHo) index of spontaneous neural activity in blood oxygen level-dependent (BOLD) signals. ReHo is a data-driven metric that reflects regional synchronization of a time series between an individual voxel and its neighboring voxels (Zang et al., 2004). ReHo analysis has been used in studies of various emotional and psychiatric illnesses, including depression (Lai and Wu, 2016), schizophrenia (Li et al., 2014), attention-deficit/hyperactivity disorder (Shang et al., 2018), and CD (Wu et al., 2017).

In this study, we conducted ReHo analysis of a rs-fMRI signal, to compare spontaneous neuronal activity between EO-CD and AO-CD. Our aim was to obtain new neuroimaging evidence to understand the pathological mechanisms that underly different subtypes of CD, thus contributing to optimizing the reliability of a clinical diagnosis, the evaluation of therapeutic efficacy, and targeted interventions of CD. We hypothesized that ReHo from rs-fMRI would reveal distinctive characteristics of spontaneous brain activity among CD subtypes, consistent with the developmental taxonomic theory.

## Materials and Methods

### Participants

We recruited 36 EO-CD and 32 AO-CD male patients, which were aged 12–16 years, from out-patient clinics affiliated with the Second Xiangya Hospital of Central South University (Changsha, Hunan, China). Diagnoses of CD were affirmed by two well-trained psychiatrists based on the Structural Clinical Interview for DSM-IV-TR Axis I Disorder-Patient Edition (SCID-I/P; First et al., 2001). According to the DSM-IV-TR, patients who exhibited at least one symptom of CD prior to being 10 years old were diagnosed with EO-CD. If the onset of symptoms was after the child’s 10th birthday, a diagnosis of AO-CD was made. To improve diagnosis reliability, detailed information was obtained from the patient and at least one corresponding parent. If the information provided by the patient was inconsistent with that obtained by the parents, the psychiatrists made the final judgment. A group of 30 age- and IQ-matched healthy controls (HCs) were recruited from local middle schools in Changsha. None of the HC volunteers met the SCID-I/P diagnostic criteria for any psychiatric disorders, including CD, or had a history of CD symptoms or aggression.

The exclusion criteria of the CD groups and HCs were: a history of attention-deficit/hyperactivity disorder, any pervasive developmental disorder (e.g., autism spectrum disorder), any psychiatric or emotional disorder (e.g., post-traumatic stress disorder), any chronic neurological disorder (e.g., obsessive compulsive disorder, Tourette’s syndrome), head trauma, persistent headaches, alcohol/substance abuse in the past year, contraindications to MRI, or an IQ ≤80 obtained with the Chinese version of the Wechsler Intelligence Scale for Children (C-WISC; Gong and Cai, 1993). All participants were right-handed, as indicated by the Edinburgh Handedness Inventory (Oldfield, 1971).

The study protocol was approved by each participating school’s administration and the Ethics Committee of the Second Xiangya Hospital of Central South University. All subjects and their parents were informed of the study’s purpose, and each participant’s parents provided written informed consent.

### Self-Report Assessments

All participants completed the Barratt Impulsivity Scale-version 11 (BIS-11) to evaluate impulsivity. In addition to the total scores, three subtypes of BIS scores were measured: attention impulsivity (attention and cognitive instability dimensions), motor impulsivity (motor and perseverance dimensions) and unplanned impulsivity (self-control and cognitive complexity dimensions; Yao et al., 2007). The Antisocial Process Screening Device (APSD) was used to assess callous unemotional traits (Vitacco et al., 2003). These scales have shown good reliability and validity in previous CD studies (Zhang et al., 2014; Dong et al., 2017; Cao et al., 2018; Sun et al., 2018b).

### MRI Acquisition

All rs-fMRI data were acquired on a Philips Achieva 3.0-T scanner using an echo-planar imaging (EPI) sequence. The scan parameters were: repetition time/echo time = 2,000/30 ms; flip angle = 90°; matrix = 64 × 64 pixels; field of view = 240 × 240 mm^2^; thickness/gap = 4.0/0 mm; number of volumes = 206; and resting acquisition time = 6 min 52 s. Head movement and noise were reduced by padding around the head and fitting ear plugs respectively.

### Data Preprocessing

Data preprocessing was conducted with the Data Processing Assistant for Resting State fMRI software^1^ (Yan and Zang, 2010). The first 10 time points for each subject were discarded to alleviate the effects of participant adaption and signal instability. For each image, slice timing was applied with the middle slice, as the reference slice and realignment for motion correction was applied. Friston 24-parameter head-motion modeling, mean white matter and cerebrospinal fluid were conducted as additional nuisance covariates. The corrected images were normalized to a standard EPI template with a voxel size of 3 × 3 × 3 mm^3^, in accordance with well-established methods for fMRI studies (Liu et al., 2013; Michalska et al., 2016; Wu et al., 2017). Signal linear detrending and band-pass filtering of data with residual signals within the range of 0.01–0.08 Hz were applied to remove biases from physiological high-frequency noise and low-frequency drift. Participants whose head motion exceeded 2 mm in translation or 2° in rotation were excluded (Sun et al., 2018a). We calculated the mean frame-wise displacement (FD) for three groups (Power et al., 2012), and found that there were no significant inter-group differences in the mean FD (Table 1). None of the participants had head motion exceeding two standard deviations (SDs) from the mean FD (Satterthwaite et al., 2013).

**Table 1:** Comparison of demographic and psychometric characteristics of the EO-CD, AO-CD, and HC groups.

	Group (mean ± SD)		
Variable	a. EO-CD	b. AO-CD	c. HC	*F* value	*P* value	*Post hoc* results
	(*N* = 36)	(*N* = 32)	(*N* = 30)			
Age, years	14.33 ± 1.29	14.78 ± 0.91	14.77 ± 1.17	1.71	0.19	
C-WISC	104.20 ± 6.30	103.55 ± 8.58	106.95 ± 6.46	1.96	0.15	
FD	0.20 ± 0.08	0.18 ± 0.06	0.18 ± 0.06	0.86	0.43	
BIS scores						
Total	76.31 ± 9.15	71.78 ± 8.10	67.40 ± 7.37	9.46	<0.001**	c < b < a
*Impulsivity subscales*						
Attention	19.21 ± 3.04	17.91 ± 2.83	18.40 ± 3.30	1.57	0.21
Motor	25.52 ± 4.24	24.44 ± 3.83	21.73 ± 3.71	7.79	0.001**	c < (a, b)
Unplanned	31.58 ± 4.21	29.44 ± 4.12	27.33 ± 3.78	9.02	<0.001**	c < b < a
APSD-CU	6.39 ± 2.19	5.28 ± 1.76	4.03 ± 1.63	12.66	<0.001**	c < b < a

EO-CD, early-onset conduct disorder; AO-CD, adolescent-onset conduct disorder; HC, healthy control; SD, standard deviation; C-WISC, Chinese Wechsler Intelligence Scale for Children; FD, frame-wise displacement; BIS, Barratt Impulsive Scale; APSD-CU, Antisocial Process Screening Device-Callous Unemotional. **p < 0.001.

### ReHo Analysis

We generated a ReHo map for each participant by calculating a Kendall’s coefficient of concordance of the time series of a given voxel, with its nearest 26 neighboring voxels (Zang et al., 2004) using the rs-fMRI Data Analysis Toolkit, version 1.8^2^ (Song et al., 2011). For standardization purposes, each subject’s ReHo map was divided by the mean whole brain ReHo. The standardized ReHo images were smoothed with a Gaussian filter of 6 mm full-width at half-maximum.

### Statistical Analysis

One-way analysis of variance (ANOVA) were used to assess inter-group variance in demographic and clinical characteristics using the Statistical Package for Social Sciences for Windows 16.0 (SPSS Inc., Chicago, IL, USA; Villers Ruiz, 1981). One-sample *t*-tests were conducted within each group to detect which ReHo values were significantly larger than the global mean ReHo value. The results were corrected by the false discovery rate (FDR), *p* < 0.05. One-way analysis of covariance (ANCOVA) was used to identify the differences of ReHo values between the EO-CD, AO-CD, and HCs groups with their age and IQ as covariates in SPM8^3^. Regions found to have significant ReHo differences among the three groups were extracted as regions of interest and *post hoc* two-sample *t*-tests were performed to detect differences in mean ReHo values between the EO-CD, AO-CD, and HC groups. Statistical test result thresholds were set to *p* < 0.05 (FDR corrected); the individual voxel-wise threshold was *p* < 0.005 with a 30-voxel extension threshold.

## Results

### Demographic and Clinical Characteristics

The three groups showed insignificant differences in age, IQ and FD (*p* > 0.05), as detailed in Table 1. The study groups differed in BIS total scores, BIS motor/non-planned subscale scores, and callous unemotional trait scores (*F* = 9.46, *p* < 0.001; *F* = 7.79, *p* < 0.001; *F* = 9.02, *p* < 0.001; *F* = 12.66, *p* < 0.001, respectively), with CD subtypes reporting significantly higher scores than HCs on these four measures (*p* < 0.001). All of these scores except the motor impulsivity sub score were significantly higher in the EO-CD group than in the AO-CD group (*p* < 0.05).

### ReHo

**Figure 1:**
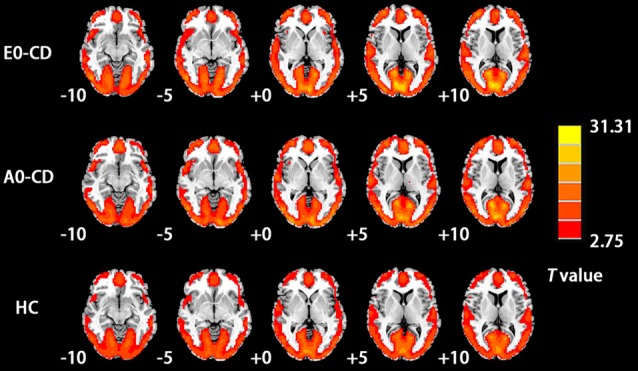
Mean regionalhomogeneity (ReHo) maps for the early-onset conduct disorder (EO-CD), adolescent-onset-CD (AO-CD), and healthy control (HC) groups. Significance was determined by one-sample *t*-tests, corrected by false discovery rate (FDR; *p* < 0.05). The left side of the figure corresponds to the right side of brain.

The mean ReHo maps for each group are presented in Figure 1 (*p* < 0.05, FDR corrected). Notably, compared to the global average ReHo value, ReHo values were significantly higher in several default mode network (DMN) regions, including the precuneus, medial prefrontal cortex, and the inferior parietal lobe.

**Figure 2:**
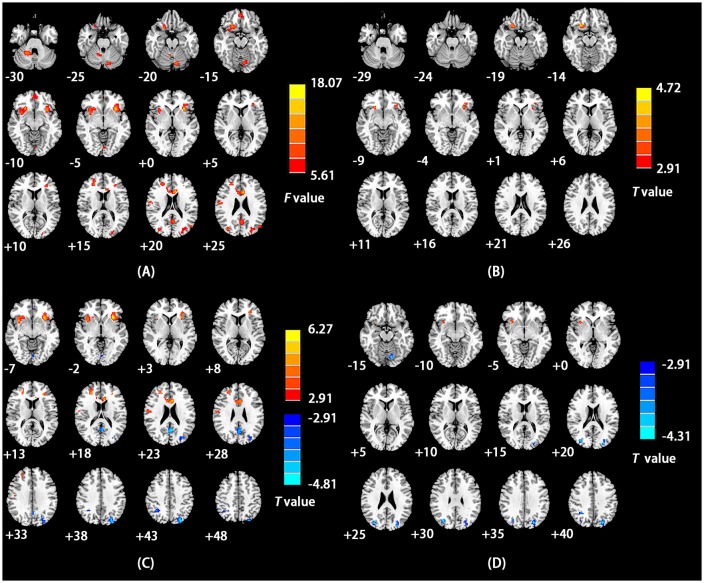
**(A)** Brain regions with a main effect of the group on ReHo values. The color bars signify *F* values. **(B)** Brain regions showing significant differences in ReHo values between the EO-CD and AO-CD groups. Warm indicates regions with increased ReHo in EO-CD vs. AO-CD. **(C)** Brain regions with significant differences in ReHo values between EO-CD patients and HCs. Warm and cool colors, respectively, indicate increased and decreased ReHo in EO-CD patients compared with HCs. **(D)** Brain regions with significant differences in ReHo values between AO-CD and HCs. Cool colors indicate decreased ReHo in AO-CD patients compared with HCs. For all panels, the left side of the figure corresponds to the right side of the brain.

As shown in Table 2 and Figure 2A, using ANCOVA, ReHo was found to differ significantly among study groups in the right middle frontal gyrus, left inferior frontal gyrus/insula, left precuneus, bilateral cerebellum posterior lobe, right inferior parietal lobule, right precentral gyrus and the left anterior cingulate cortex.

**Table 2:** Brain regions with ReHo differences between the EO-CD or AO-CD group and the HC group.

Brain region	Cluster size (voxels)	MNI coordinates			Peak *F* value
		*X*	*Y*	*Z*	
R middle frontal gyrus	85	27	39	15	10.79
L inferior frontal gyrus/insula	162	−30	21	−3	18.07
R cerebellum posterior lobe	163	21	−48	−33	13.84
L cerebellum posterior lobe	80	−9	−78	−21	11.32
L precuneus	174	−27	−81	45	13.84
R inferior parietal lobule	47	33	−54	42	10.97
R precentral gyrus	40	57	−9	24	12.03
L anterior cingulate cortex	94	−6	18	24	15.88

*ReHo, regional homogeneity; EO-CD, early-onset conduct disorder; AO-CD, adolescent-onset conduct disorder; HC, healthy control; MNI, Montreal Neurological Institute; L, left; R, right*.

The EO-CD group showed higher ReHo values in the right middle/inferior frontal gyrus relative to the AO-CD group (Table 3 and Figure 2B). Relative to HCs, the EO-CD group had lower ReHo values in the left precuneus, left middle occipital gyrus, left cerebellum posterior lobe and the right inferior parietal lobule, as well as higher ReHo values in the right middle frontal gyrus, left insula/inferior frontal gyrus, right precentral gyrus and the left anterior cingulate cortex (Table 3 and Figure 2C). Compared to HCs, the AO-CD group had lower ReHo values in the bilateral precuneus, left cerebellum posterior lobe and the right inferior parietal lobule (Table 3 and Figure 2D).

**Table 3:** Comparisons of ReHo values among EO-CD or AO-CD and HCs groups.

Brain region	Cluster size (voxels)	MNI coordinates			Peak *t* value
		*X*	*Y*	*Z*	
**EO-CD vs. AO-CD**					
*EO-CD > AO-CD*					
R middle/inferior frontal gyrus	59	24	21	−12	4.72
**EO-CD vs. HC**					
*EO-CD < HC*					
L precuneus	77	−3	−57	21	−4.75
L middle occipital gyrus	170	−27	−81	45	−4.81
L cerebellum posterior lobe	71	−6	−78	−18	−4.74
R inferior parietal lobule	39	36	−54	42	−3.96
*EO-CD > HC*					
R middle frontal gyrus	85	27	39	15	4.84
L insula/inferior frontal gyrus	153	−30	21	0	6.27
R postcentral gyrus	36	54	−9	24	4.04
L anterior cingulate cortex	94	−6	18	24	5.52
**AO-CD vs. HC**					
*AO-CD < HC*					
L precuneus	128	−24	−78	39	−4.29
R precuneus	53	33	−72	30	−4.31
L cerebellum posterior lobe	52	−12	−78	−18	−4.04
R inferior parietal lobule	43	36	−54	45	−4.01

*EO-CD, early-onset conduct disorder; AO-CD, adolescent-onset conduct disorder; HCs, healthy controls; L, left; R, right*.

## Discussion

To our knowledge, this is the first study to explore regional synchronization abnormalities of brain spontaneous activity in EO-CD and AO-CD patients. Our results demonstrated the EO-CD had more severe symptoms, and higher ReHo in the right middle/inferior frontal gyrus compared with AO-CD, which were consistent with the developmental taxonomic theory.

The EO-CD group had higher ReHo values compared to the AO-CD group in the right middle/inferior frontal lobe. These findings are particularly interesting given that frontal lobe lesions have been shown to be associated with the presence of CD symptoms (Pennington and Bennetto, 1993). In a study of patients with middle frontal gyrus lesions, Aron et al. (2004a) found that the degree of damage affecting the left middle frontal gyrus correlated closely with the severity of top-down control weakening observed. The middle frontal gyrus has been regarded as a core element of the executive attention network regulating emotional behavior and cognition (Schilling et al., 2012; Kogler et al., 2015). The right inferior frontal gyrus is implicated in impulse control and risk aversion (Aron et al., 2004b; Guo et al., 2012). The middle/inferior frontal gyrus hyperactivation has been seen during affective processing and moral decision tasks in other study groups, such as high-psychopathy community members and criminals (Kiehl et al., 2001; Marsh and Cardinale, 2014).

CD patients have been reported to be at elevated risk of psychopathic traits (Barry et al., 2000). Elevated ReHo values in the middle/inferior frontal gyrus in individuals with CD point to abnormal enhanced synchronization patterns that may affect emotion and cognition. Higher ReHo values in the middle frontal gyrus in EO-CD patients, relative to AO-CD patients, may be associated with more severe cognitive and emotional deficits, which support the developmental taxonomic theory (Moffitt, 1993). Impaired baseline spontaneous brain activity in the middle/inferior frontal gyrus should be investigated as a potential early biomarker of a functional abnormality in EO-CD patients (Qiao et al., 2012; Dalwani et al., 2014). It might reflect that these deficits are specific features of EO-CD.

Compared with HCs, both CD subtypes had decreased ReHo in the bilateral precuneus, right inferior parietal lobule and the left cerebellum posterior lobe. The precuneus and inferior parietal lobule are known as components of the DMN, a key network for self-referential experiences processing, social perspective-taking, and the construction of self and others’ future possible thoughts and behaviors (Yao et al., 2009; Andrewshanna, 2012; Whitfield-Gabrieli and Ford, 2012). Individuals who are unable to be properly responsible for negative consequences, tend to engage in rule-breaking and antisocial behavior (Raine and Yang, 2006). The fact that ReHo values in the precuneus and inferior parietal lobule were altered in CD patients, supports the view that abnormal DMN activity may be secondary to CD. In line with these findings, prior rs-fMRI studies have reported abnormal neural activity in the DMN as well as reduced functional connectivity within the DMN and between DMN regions and other brain regions in CD patients (Broulidakis et al., 2016; Zhou et al., 2016; Wu et al., 2017).

The cerebellum, which is connected to the prefrontal cortex and limbic regions (hippocampus and amygdala), has been implicated in high-order cognition and emotion, in addition to motor coordination and control (Schmahmann and Caplan, 2006). Notably, the posterior lobe of the cerebellum has been associated with cognitive and affective deficits, whereas the posterior vermis has been shown to play a pivotal role in neuropsychiatric impairments and the anterior lobe has been shown to be involved in processing sensorimotor information (Stoodley and Schmahmann, 2010). Abnormal spontaneous activity in the DMN and posterior lobe of the cerebellum might influence patient’s psychological processing, and thereby may contribute to the abnormal callous-unemotional trait and antisocial behavior characteristics of CD. Further research is required to confirm this finding.

Compared with HCs, EO-CD showed increased ReHo activity in the left anterior cingulate gyrus and the right postcentral gyrus. In an affective stimulation task, the anterior cingulate gyrus was found to be associated with impulsivity in CD patients (Stadler et al., 2007). The postcentral gyrus, located in the lateral parietal lobe, is the primary somatosensory area of the cerebral cortex (Kaas and Collins, 2001). The somatosensory networks are regarded as constituting low-level perceptual systems. The present findings, suggestive of abnormal somatosensory networks in CD, are in line with a previous resting state study in which CD patients had dysfunction in the DMN as well as in low-level perceptual systems (Lu et al., 2015).

This study had several limitations. First, like most neuroimaging studies of mental disorders, the study was cross-sectional. Future longitudinal studies are needed to investigate EO-CD and AO-CD. Second, in the future multi-parametric neuroimaging methods should be applied to explore the pathophysiology in both CD subtypes with larger samples, in order to further validate our findings. Finally, because this study included only male participants, our findings may not generalize to female adolescents.

## Conclusion

The present analysis of the ReHo index of spontaneous neuronal activity revealed significant differences in baseline brain activity between patients with EO-CD vs. AO-CD, suggesting that these two CD subtypes may have different neuropathological mechanisms, consistent with the developmental taxonomic theory. The areas of abnormal neuronal synchronization of spontaneous brain activity associated with EO-CD and AO-CD distributed mainly in the frontal lobe, DMN, cerebellum, anterior cingulate gyrus, visual network, and the somatosensory network. These alterations may be associated with impairments related to high-order cognitive networks and low-level perceptual systems in CD patients.

## Author Contributions

SY, BH and JL conceived and designed the study. WC, CL, JZ, DD and XS were involved in the data collection and analyses. DD and XS helped to draft the manuscript. WC and JZ wrote the manuscript.

## Conflict of Interest Statement

The authors declare that the research was conducted in the absence of any commercial or financial relationships that could be construed as a potential conflict of interest.

## Footnotes

^1^http://rfmri.org

^2^http://restfmri.net/forum/REST

^3^http://www.fil.ion.ucl.ac.uk/spm
